# Identifying Subgroups in Acceptance and Intended Use of Digital Technologies and the Role of Economic, Cultural, Social, and Person Capital: Latent Class Analysis

**DOI:** 10.2196/89446

**Published:** 2026-09-24

**Authors:** Wendy Wagenaar, Marieke Christina van Egmond, Joyce Bierbooms

**Affiliations:** 1 Tranzo, Scientific Center for Care and Wellbeing, Tilburg School of Social and Behavioral Sciences, Tilburg University, Prof. Cobbenhagenlaan 125, Tilburg, 5037 DB, The Netherlands, +31 13 466 2969; 2 Academic Collaborative Center for Digital Health & Mental Wellbeing, Tilburg University, Tilburg, The Netherlands

**Keywords:** digital health, digital divide, socioeconomic factors, digital technology, social inequality

## Abstract

**Background:**

Digital health technologies offer promising opportunities to support physical health. However, their acceptance, use, and associated benefits are not equally distributed across society. While existing research has mainly focused on traditional socioeconomic indicators, broader sociological influences, including economic, cultural, social, and person capital, may provide a more comprehensive understanding of these inequalities. Yet, too little is currently known about how different subgroups, based on their economic, cultural, social, and person capital, relate to intentions to accept and use digital health technologies.

**Objective:**

This study aimed (1) to identify distinct subgroups of individuals based on their acceptance and intended use of digital technologies to support their physical health and (2) to examine how these subgroups differ in terms of economic, cultural, social, and person capital.

**Methods:**

We used cross-sectional data from the Longitudinal Internet Studies for the Social Sciences (LISS) panel, including data from the LISS Core Study on health, economic situation, and social integration and leisure. To supplement these data, we conducted an additional online survey in November 2023 via the LISS panel to assess participants’ acceptance and intended use of digital technologies to support their physical health. The final sample included 1096 participants. We applied 3-step latent class analysis to identify subgroups based on constructs from the unified theory of acceptance and use of technology. Post hoc comparisons were used to characterize the subgroups based on 22 indicators of economic, cultural, social, and person capital.

**Results:**

Five subgroups were identified: neutral users (480/1096, 43.8%), uninterested users (235/1096, 21.4%), engaged users (226/1096, 20.6%), resistant users (102/1096, 9.3%), and enthusiastic users (53/1096, 4.8%). The largest group, neutral users, neither fully adopted nor rejected digital technologies to support their physical health. Higher levels of economic, cultural, and social capital were generally associated with greater acceptance and intended use of digital health technologies. However, person capital showed a different pattern: neutral users reported low self-confidence despite moderate use, while resistant users reported high self-image despite low acceptance and intended use. This suggests that person capital relates to the acceptance and intended use of digital health technologies in a different way than economic, cultural, and social capital.

**Conclusions:**

Inequalities in digital health engagement extend beyond socioeconomic factors and reflect broader differences in economic, cultural, social, and person capital. The distinct user types that were identified reveal that combinations of different types of capital can influence acceptance and intended use in unexpected ways. Addressing these multidimensional disparities is crucial for designing targeted and equitable strategies to enhance digital health participation across diverse populations.

## Introduction

In recent years, the use of digital health technologies to support physical health has grown rapidly, offering promising opportunities for both patients and health care professionals. These technologies range from websites, mobile health apps, and wearable fitness trackers to even gaming consoles [1-4]. For patients, digital health technologies can support self-management [5-7], encourage increased physical activity [2,8], and contribute to physical and mental well-being [9,10]. For health care professionals, these tools offer opportunities to enhance diagnosis and treatment [6,11], as well as improve communication with patients [12,13]. However, despite their potential, the benefits of digital health technologies are not equally distributed across society.

A growing body of research has highlighted how digital health technologies may exacerbate existing health inequalities in society, due to substantial differences in both access and use of these technologies [14-17]. The gap between different social groups in relation to digital health technologies is commonly referred to as the “digital divide” [16,18]. This divide includes not only issues of access (eg, the accessibility divide) but also differences in how technologies are used and who ultimately benefits from them (eg, the usability divide) [19-21]. When designing and implementing digital health technologies, it becomes increasingly important to address this digital divide and the underlying societal inequalities that contribute to it.

To explore acceptance and intended use of digital health technologies across the digital divide, many studies have looked into the role of socioeconomic indicators. For example, education has consistently been found to play a key role, with individuals with a higher educational level being more digitally engaged and more likely to benefit from digital health technologies [22-24]. Similarly, income and age have been found to significantly influence acceptance and intended use of digital health technologies; individuals with a higher income and those who are younger are generally more likely to access and effectively use digital health technologies [25-29]. While these socioeconomic indicators provide valuable insights on the acceptance and intended use of digital health technologies, it has recently been argued that a more holistic approach is needed, one that goes beyond traditional socioeconomic measures and considers a broader range of sociological influences [30].

This holistic approach to inequalities for health technologies distinguishes 4 forms of capital: economic, social, cultural, and person capital [31,32]. Economic capital refers to resources such as education, income, and financial assets [33], which may influence the use of digital health technologies by providing the financial means or opportunities to access and use them. Social capital relates to an individual’s relationships and position within social networks, which can provide both social support (eg, someone to talk to or do activities with) and instrumental support (eg, help with administration) [31,34,35]. These social networks may influence technology use if individuals observe others in their network using it or if they can request (technical) support. Cultural capital includes knowledge, (digital) skills, and access to cultural resources, including digital skills, attending theater, or international travel [31,36,37]. Finally, person capital, which is a rather novel concept in terms of capital, includes aspects of physical and mental health, well-being, and aesthetic appearance that may affect a person’s societal position [31]. This includes physical indicators, such as self-rated health, long-term health limitations, and BMI, as well as mental and self-perception indicators, such as depressive symptoms, self-confidence, and self-image [32]. These indicators are found to construct someone’s ability to gain social advantage and are therefore important metrics to operationalize the concept of person capital [38]. Integrating these 4 forms of capital provides a more comprehensive understanding of social inequalities in access and use of digital health technologies, moving beyond traditional socioeconomic indicators.

Based on existing literature, we can hypothesize that variations in individuals’ economic, cultural, social, and person capital each relate to the accessibility and usability of digital health technologies in different ways [30]. Yet, too little is currently known about these 4 forms of capital and their relation to individuals’ intentions to accept and use digital health technologies [30]. While some studies have examined the impact of specific indicators, such as social connections [24] and digital skills [39], a comprehensive understanding of the role of all 4 types of capital on the acceptance and intended use of digital technologies remains lacking. Understanding these 4 forms of capital may provide an interdisciplinary approach to technology adoption by complementing established technology adoption models, such as the technology acceptance model and the unified theory of acceptance and use of technology (UTAUT) [40,41]. The 4 forms of capital may help explain the broader economic, cultural, social, and personal factors that shape the determinants of technology adoption, thereby influencing patterns of acceptance and intended use across population subgroups. To move toward digital health technologies that do not exacerbate existing health inequalities, it is essential to better understand which subgroups exist based on their acceptance and intended use of digital health technologies and how different types of capital characterize these subgroups. As digital inequalities may not be reflected by a single indicator alone, identifying these subgroups can extend existing research by showing which groups may require different forms of support, such as digital skills training or targeted onboarding. This knowledge can help researchers and practitioners develop more tailored and effective strategies to increase the acceptability and usability of digital health technologies across all societal groups.

Therefore, this study aims (1) to identify distinct subgroups of individuals based on their acceptance and intended use of digital technologies to support their physical health and (2) to examine how variations in economic, cultural, social, and person capital are associated with these subgroups.

## Methods

### Participants and Procedures

To identify subgroups based on acceptance and intended use of digital technologies to support their physical health, we conducted a cross-sectional study using data from the Longitudinal Internet Studies for the Social Sciences (LISS) panel. The LISS panel is administered and managed by the nonprofit research institute Centerdata (Tilburg University, the Netherlands) and consists of a representative sample of Dutch individuals of approximately 7500 from 5000 households. The panel is based on a true probability sample of households drawn from the population register by Statistics Netherlands. Self-registration is not possible, and households who would otherwise be unable to participate are provided with a computer and internet connection.

A standard component of the LISS panel is the LISS Core Study, which has been conducted in the panel every year since 2007, covering a large variety of domains including health, work, education, income, housing, leisure and time use, political views, values, and personality. For our study, we used existing data from the LISS Core Study in the domains of health [42] (November or December 2023 wave), economic situation [43] (June or July 2023 wave), and social integration and leisure [44] (October or November 2023 wave).

To supplement the data from the LISS Core Study, we conducted an additional survey in November 2023 to specifically assess the acceptability and use of digital technologies, along with relevant individual characteristics of the 4 types of capital. A random sample of 1450 panel members was invited to complete the additional survey, resulting in 1107 responses (response rate 76.3%). After excluding 11 cases due to missing data on key variables used to construct composite constructs, the final sample included 1096 participants (75.6% of those invited).

The data from the LISS Core Study and the supplementary survey were merged using the unique panel member identification number to create the final dataset for the analysis.

### Ethical Considerations

This study was approved by the ethics review board of Tilburg University (reference: TSB_RP2513). To minimize the burden for participants, we primarily used existing panel data and collected additional data only where necessary.

### Measures

Acceptance and intended use of digital technologies for physical health were measured using constructs from the UTAUT model [40]. The UTAUT model theorizes that users’ intention to use and actual use of technology are influenced by several key factors. In the initial UTAUT model, behavioral intention to use technology is shaped by performance expectancy (belief that the technology will achieve the expected outcomes), effort expectancy (perceived ease of use), social influence (impact of social environment), and facilitating conditions (availability of infrastructure to use the technology) [45]. In a revised version of the model, 3 additional factors were included: hedonic motivation (enjoyment or pleasure from using the technology), habit (extent to which technology use is automatic or routine), and price value (perceived trade-off between the benefits and costs of using the technology) [40]. For this study, items of the UTAUT model were adapted from prior studies [40,46] and tailored to the context of digital technologies for physical health. The full list of UTAUT items used in this study is provided in Table S1 in Multimedia Appendix 1. The UTAUT constructs performance expectancy, effort expectancy, and social influence were each assessed using multiple items. Based on factor and reliability analyses, 2 items were excluded from the final scales: item 3 from effort expectancy and item 4 from social influence. The final internal consistency of the multi-item constructs as measured by Cronbach α was α=0.872 for performance expectancy, α=0.860 for effort expectancy, and α=0.688 for social influence. Additional constructs from the UTAUT model, facilitating conditions, hedonic motivation, habit, and price value, were each measured using a single item. Although multi-item measures are generally preferable because they allow reliability to be assessed and may reduce measurement error, these constructs were measured using single items due to strict space limitations in the LISS supplementary survey and to reduce respondent burden.

To measure individual characteristics associated with the 4 types of capital, we used indicators based on the framework developed by the Sociaal Cultureel Planbureau (Netherlands Institute for Social Research). This is a set of 18 indicators covering the 4 types of capital, as proposed by Vrooman et al [38]. These indicators have been priorly used to identify subgroups of capital to address substantial structural differences in Dutch society, but are also relevant in our context because they capture the dimensions of capital that may influence the acceptance and intended use of digital health technologies. Using these established indicators also facilitates the comparison and potential generalization of our findings to other studies. To collect data, wherever possible, these indicators were drawn from existing data in the LISS Core Study. Indicators not available in the existing dataset were included in the additional survey. Based on reliability analyses, several items were split into separate indicators (eg, the lifestyle construct was divided into “international holidays,” “frequency of attending concerts, theater, and museums,” and “use of sustainable food”), resulting in a total of 22 capital indicators. An overview of the specific items used for the capital indicators is listed in Table S2 in Multimedia Appendix 1.

Sociodemographic variables, including sex, age, and origin, were derived from the LISS Core Study: Background Variables [47]. The specific items used for the sociodemographic variables are provided in Table S3 in Multimedia Appendix 1.

### Statistical Analysis

To identify subgroups based on their acceptance and intended use of technology for physical health, we conducted a 3-step latent class analysis using Latent GOLD (version 6.1) [48].

In step 1, we estimated latent class models using constructs from the UTAUT framework [49,50]. Missing data were kept in the analysis using full information maximum likelihood estimation under the assumption that data were missing at random. To determine the optimal number of latent classes, we evaluated model fit using the Bayesian information criterion (BIC), Akaike information criterion (AIC), log-likelihood (LL), and the maximum bivariate residual (BVR). For model selection, lower values of BIC and AIC indicate better model fit [51-53]. In addition, BVR values below 3.84 suggest no significant covariation between indicator pairs [54]. However, as models with too many classes may become difficult to meaningfully interpret [48], model interpretability was also considered. Therefore, rather than solely examining absolute statistics for model fit, we assessed improvements in LL, AIC, and BIC across models with increasing numbers of classes, relative to the improvement between the 1- and 2-class models (ie, relative LL, AIC, and BIC). This approach follows the latent class tree modeling strategy proposed by Van Den Bergh and Vermunt [55]. Similarly, we examined the relative change in maximum BVR values compared to the 1-class model (ie, relative maximum BVR).

In step 2, we explored associations between the identified latent classes and the indicators related to the 4 types of capital. Posterior membership probabilities were obtained using proportional assignment, the default in Latent GOLD [48]. These posterior probabilities were subsequently used in step 3 to account for classification error.

In step 3, we tested whether class membership was significantly associated with capital indicators using pairwise Wald tests. For nominal variables, we applied the maximum likelihood method [56], and for continuous variables, the Bolck-Croon-Hagenaars method [57]. We identified significant associations by evaluating differences between classes, where differences were considered statistically significant at *P*<.05. As a sensitivity check, we adjusted the *P* values from the pairwise Wald tests using the Benjamini-Hochberg false discovery rate correction to examine the robustness of the pairwise class differences. In addition, we repeated the step 3 analyses separately for participants aged 55 years and younger and those aged 55 years or older, while retaining the same 5-class solution. Finally, we conducted descriptive analyses using SPSS Statistics (version 29.0; IBM Corp).

## Results

### Model Selection

Table 1 shows the model fit statistics ranging from 1 to 10 classes. Based on the absolute values of the LL, BIC, and AIC, the 9-class model showed the best fit. However, models with a larger number of classes may become increasingly difficult to interpret meaningfully [48]. Therefore, we also examined the relative improvement in model fit across the classes. The relative improvement of fit decreased from 0.10 to 0.08 (relative LL), from 0.07 to 0.05 (relative BIC), and from 0.09 to 0.07 (relative AIC) when moving from 5 to 6 classes and relatively stagnated thereafter. This showed that adding one extra class beyond the 5-class model did not yield large fit improvements. In addition, the relative maximum BVR confirmed that the 5-class model sufficiently captured the key associations among the class indicators. The 5-class model also showed an entropy value of 0.80, suggesting adequate classification quality. Therefore, the 5-class model was selected.

**Table 1.** Model fit statistics for 1- to 10-class solutions.

Model	LL^a^	BIC^b^	AIC^c^	Maximum BVR^d^	*P* value BLRT^e^	Npar^f^	Entropy *R*^2^	Rel^g^ LL	Rel BIC	Rel AIC	Rel maximum BVR
1-class	−12,879.7	26,095.4	25,855.5	714.19	—^h^	48	—	—	—	—	—
2-class	−11,927.1	24,246.1	23,966.1	138.33	<.001	56	0.83	—	—	—	—
3-class	−11,435.9	23,319.8	22,999.8	43.07	<.001	64	0.86	0.52	0.50	0.51	1.17
4-class	−11,271.0	23,046.0	22,686.1	16.71	<.001	72	0.81	0.17	0.15	0.17	1.21
5-class^i^	−11,178.8	22,917.5	22,517.5	15.27	<.001	80	0.80	0.10	0.07	0.09	1.21
6-class	−11,106.3	22,828.6	22,388.7	12.82	<.001	88	0.82	0.08	0.05	0.07	1.22
7-class	−11,080.8	22,833.6	22,353.7	12.57	.008	96	0.81	0.03	0.00	0.02	1.22
8-class	−11,059.4	22,846.8	22,326.9	11.08	.008	104	0.80	0.02	−0.01	0.01	1.22
9-class	−11,038.9	22,861.6	22,301.7	10.87	.01	112	0.80	0.02	0.00	0.01	1.22
10-class	−11,022.2	22,884.3	22,284.3	10.52	.03	120	0.79	0.02	−0.01	0.01	1.22

a LL: log-likelihood.

b BIC: Bayesian information criterion.

c AIC: Akaike information criterion.

d BVR: bivariate residual.

e BLRT: bootstrap likelihood ratio test.

f Npar: number of parameters.

g Rel: relative difference.

h Not available.

i Selected model.

### Class Description

**Figure 1.**
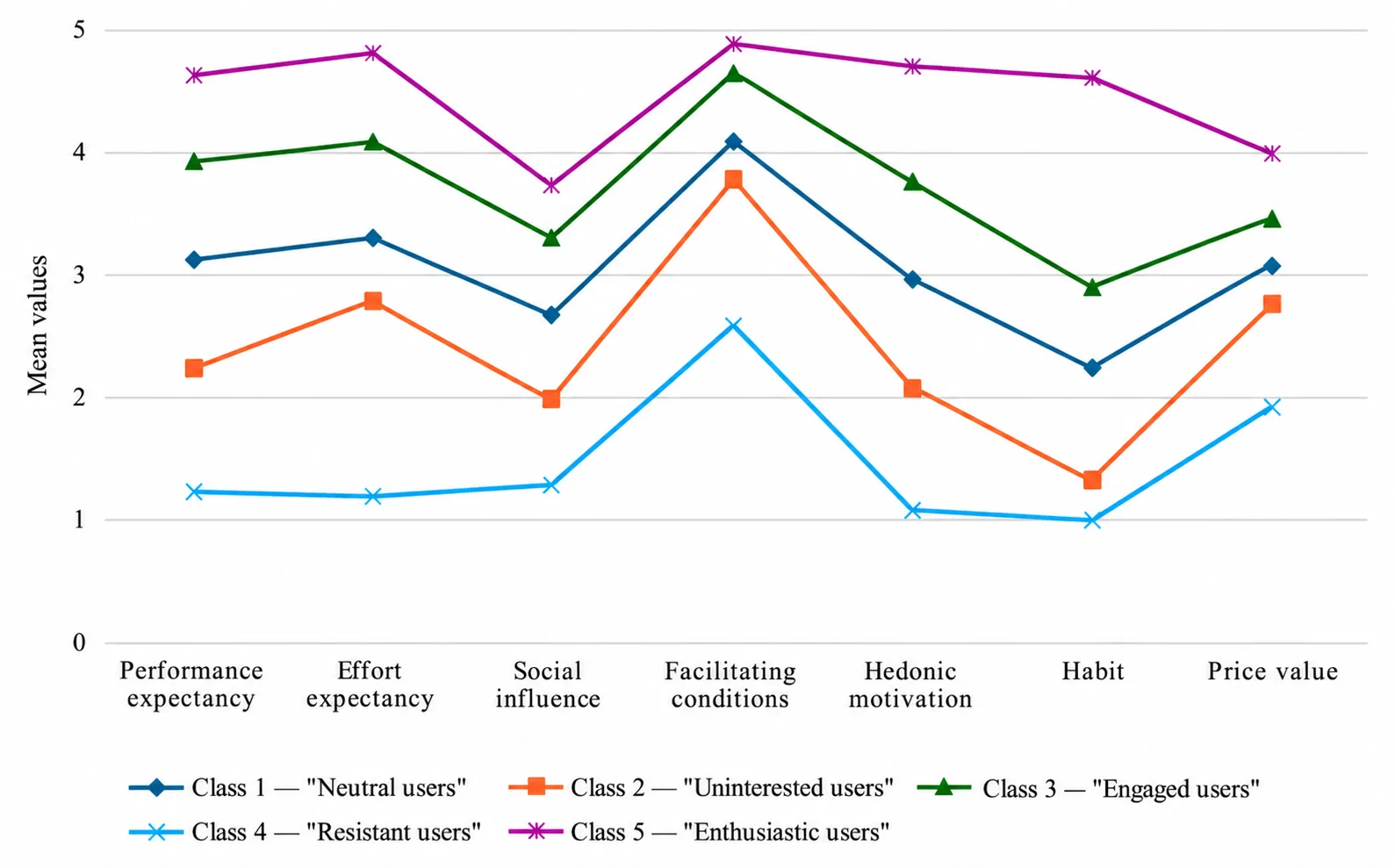
Mean scores of unified theory of acceptance and use of technology constructs across the 5 latent classes.

Figure 1 displays the mean values of all UTAUT constructs for the 5 identified classes, and Table 2 summarizes the significant differences between them. Table 3 describes the sociodemographic characteristics across the 5 latent classes. Class 1 (480/1096, 43.8% of the sample) comprised the largest subgroup, with moderate scores on all UTAUT constructs. Their scores were significantly lower than those of class 3 and 5, but higher than those of class 2 and 4. Given the relatively moderate scores across all constructs, this group was labeled “neutral users.” Class 2 (235/1096, 21.4% of the sample) consistently scored lower than class 1, 3, and 5, but higher than class 4. Their generally low scores suggest hesitant attitudes toward acceptance and intended use of digital health technologies. Therefore, they were labeled as “uninterested users.” Class 3 (226/1096, 20.6% of the sample) showed generally positive mean scores that were significantly higher than those of class 1, 2, and 4, but lower than those of class 5. This group appeared to be positive and fairly engaged and was thus labeled “engaged users.” Class 4 (102/1096, 9.3% of the sample) showed the lowest mean scores of all classes on every UTAUT construct. This indicated a strongly negative attitude toward acceptance and intended use of digital technology for their physical health. Accordingly, this group was labeled “resistant users.” Finally, class 5 (53/1096, 4.8% of the sample) showed the highest mean scores on all constructs, significantly exceeding those of all other classes. This group perceived digital health technologies as highly valuable, easy to use, and enjoyable. This group was therefore labeled “enthusiastic users.”

**Table 2.** Differences between the unified theory of acceptance and use of technology constructs across the 5 latent classes.

Class	Full sample	Class 1	Class 2	Class 3	Class 4	Class 5	Wald test	Post hoc
Class size, n (%)	1096 (100)	480 (43.8)	235 (21.4)	226 (20.6)	102 (9.3)	53 (4.8)	N/A^a^	N/A
Performance expectancy, mean (SD)	3.00 (0.98)	3.13 (0.43)	2.25 (0.52)	3.93 (0.38)	1.22 (0.33)	4.63 (0.36)	278.14^b^	1>2, 4; 2>4; 3>1, 2, 4; 5>1, 2, 3, 4
Effort expectancy, mean (SD)	3.23 (1.11)	3.30 (0.68)	2.77 (1.00)	4.08 (0.51)	1.19 (0.38)	4.81 (0.33)	383.40^b^	1>2, 4; 2>4; 3>1, 2, 4; 5>1, 2, 3, 4
Social influence, mean (SD)	2.58 (0.86)	2.68 (0.54)	1.99 (0.55)	3.30 (0.57)	1.26 (0.42)	3.74 (0.69)	372.48^b^	1>2, 4; 2>4; 3>1, 2, 4; 5>1, 2, 3, 4
Facilitating conditions, mean (SD)	4.03 (1.16)	4.08 (1.03)	3.77 (1.23)	4.64 (0.61)	2.58 (1.59)	4.88 (0.38)	131.22^b^	1>2, 4; 2>4; 3>1, 2, 4; 5>1, 2, 3, 4
Hedonic motivation, mean (SD)	2.85 (1.11)	2.97 (0.62)	2.08 (0.82)	3.76 (0.65)	1.08 (0.32)	4.70 (0.48)	324.26^b^	1>2, 4; 2>4; 3>1, 2, 4; 5>1, 2, 3, 4
Habit, mean (SD)	2.19 (1.19)	2.24 (0.91)	1.33 (0.51)	2.90 (1.04)	1.00 (0.00)	4.61 (0.53)	146.05^b^	1>2, 4; 2>4; 3>1, 2, 4; 5>1, 2, 3, 4
Price value, mean (SD)	3.03 (0.98)	3.09 (0.75)	2.75 (1.02)	3.45 (0.97)	1.93 (0.91)	3.99 (0.89)	454.04^b^	1>2, 4; 2>4; 3>1, 2, 4; 5>1, 2, 3, 4

a N/A: not applicable.

b *P*<.05.

**Table 3.** Sociodemographic characteristics across the 5 latent classes.

Sociodemographic characteristics	Total (N=1096, 100%), n (%)	Class 1 (n=480, 43.8%), n (%)	Class 2 (n=235, 21.4%), n (%)	Class 3 (n=226, 20.6%), n (%)	Class 4 (n=102, 9.3%), n (%)	Class 5 (n=53, 4.8%), n (%)
Sex (n=1096)						
Female	561 (51.2)	256 (53.3)	126 (53.6)	105 (46.5)	51 (50)	23 (43.4)
Male	534 (48.7)	224 (46.7)	109 (46.4)	121 (53.5)	50 (49)	30 (56.6)
Other	1 (0.1)	0 (0)	0 (0)	0 (0)	1 (1)	0 (0)
Age (years) (n=1096)						
15‐24	99 (9)	53 (11)	19 (8.1)	20 (8.8)	4 (3.9)	3 (5.7)
25‐34	171 (15.6)	59 (12.3)	30 (12.8)	62 (27.4)	9 (8.8)	11 (20.8)
35‐44	169 (15.4)	79 (16.5)	25 (10.6)	46 (20.4)	8 (7.8)	11 (20.8)
45‐54	178 (16.2)	97 (20.2)	31 (13.2)	27 (11.9)	13 (12.7)	10 (18.9)
55‐64	198 (18.1)	79 (16.5)	59 (25.1)	29 (12.8)	19 (18.6)	12 (22.6)
65 and older	281 (25.6)	113 (23.5)	71 (30.2)	42 (18.6)	49 (48)	6 (11.3)
Origin^a^ (n=1079)						
Dutch	830 (76.9)	365 (77.8)	185 (80.4)	156 (71.2)	81 (75.7)	43 (79.6)
First generation with Western background	60 (5.6)	26 (5.5)	7 (3)	14 (6.4)	9 (8.4)	4 (7.4)
First generation with non-Western background	65 (6.0)	25 (5.3)	13 (5.7)	14 (6.4)	9 (8.4)	4 (7.4)
Second generation with Western background	69 (6.4)	30 (6.4)	10 (4.3)	22 (10)	4 (3.7)	3 (5.6)
Second generation with non-Western background	55 (5.1)	23 (4.9)	15 (6.5)	13 (5.9)	4 (3.7)	0 (0.0)

a Due to missing values, the total number of respondents for origin is 1079. Percentages for origin are calculated based on participants with available origin data (n=1079).

The largest differences in mean values of the UTAUT constructs between classes were observed for effort expectancy (class 5: mean 4.81, SD 0.33 vs class 4: mean 1.19, SD 0.38), hedonic motivation (class 5: mean 4.70, SD 0.48 vs class 4: mean 1.08, SD 0.32), and habit (class 5: mean 4.61, SD 0.53 vs class 4: mean 1.00, SD 0.00). Price value had the smallest difference (class 5: mean 3.99, SD 0.89 vs class 4: mean 1.93, SD 0.91), though this difference was still considerable.

### Class Profiles Based on Economic, Cultural, Social, and Person Capital

To better understand the 5 classes, we analyzed differences across classes based on their economic, cultural, social, and person capital. Table 4 shows the significant differences that were found across the 5 classes.

**Table 4.** Means and comparison of capital indicators across the 5 latent classes.

Class	Full sample	Class 1 “neutral users”	Class 2 “uninterested users”	Class 3 “engaged users”	Class 4 “resistant users”	Class 5 “enthusiastic users”	Wald test	Post hoc^a^
Class size^b^, n (%)	1096 (100)	480 (43.8)	235 (21.4)	226 (20.6)	102 (9.3)	53 (4.8)	—^c^	—
Economic capital, mean (SD)								
Highest educational qualification obtained	3.21 (0.84)	3.20 (0.81)	3.15 (0.87)	3.49 (0.72)	2.66 (0.98)	3.52 (0.64)	141.26^d^	1, 2, 3, 5>4; 1>2; 3>1, 2; 5>1, 2, 3
Subjective socioeconomic status	7.13 (1.34)	7.15 (1.28)	7.10 (1.32)	7.35 (1.67)	6.66 (1.73)	7.23 (1.56)	213.70^d^	1, 3, 5>2; 1, 2, 3>4; 3, 5>1
Net household income	4030.60 (2249.78)	4121.51 (2284.35)	3817.18 (2098.75)	4371.61 (2345.94)	2891.37 (1443.02)	4790.41 (2593.23)	33.33^d^	1, 2, 3, 5>4; 3, 5>2
Cultural capital, mean (SD)								
International holidays	2.21 (1.27)	2.26 (1.29)	2.02 (1.22)	2.48 (1.30)	1.82 (1.03)	2.44 (1.35)	38.86^d^	3>2, 4
Frequency of attending concerts, theaters, and museums	0.44 (0.47)	0.45 (0.46)	0.40 (0.45)	0.56 (0.50)	0.29 (0.42)	0.49 (0.43)	14.17^d^	1>4; 3>1, 2, 4; 5>4
Use of sustainable food	1.94 (1.40)	1.93 (1.38)	1.83 (1.39)	2.26 (1.40)	1.34 (1.32)	2.40 (1.35)	42.33^d^	1, 3, 5>4; 3>2
Basic digital skills	0.76 (0.29)	0.75 (0.30)	0.76 (0.28)	0.87 (0.21)	0.56 (0.34)	0.85 (0.19)	196.24^d^	3>1, 2; 1, 2, 3, 5>4; 5>1
English language proficiency	3.05 (0.86)	3.03 (0.83)	2.99 (0.88)	3.34 (0.73)	2.50 (1.00)	3.38 (0.69)	124.67^d^	1, 2, 5>4; 3, 5>1; 3>2
Social capital, mean (SD)								
Contact with family members (not living in the same household)	1.61 (1.05)	1.61 (1.07)	1.62 (1.08)	1.53 (0.96)	1.84 (1.16)	1.45 (0.91)	25.98	—
Contact with close friends	1.75 (1.05)	1.76 (1.06)	1.81 (1.11)	—	1.97 (1.10)	1.53 (0.91)	23.58	—
Contact with neighbors and community members	2.64 (1.44)	2.60 (1.44)	2.61 (1.39)	2.80 (1.46)	2.65 (1.58)	2.45 (1.40)	18.49	—
Contact with colleagues outside working hours	3.45 (1.39)	3.47 (1.38)	3.51 (1.34)	3.45 (1.45)	3.50 (1.43)	3.07 (1.41)	10.93	—
Size of core discussion network	3.46 (1.62)	3.41 (1.62)	3.32 (1.64)	3.97 (1.43)	2.57 (1.61)	4.13 (1.32)	75.64^d^	1, 2, 3, 5>4; 3>1, 2; 5>2
Informal support	2.98 (1.24)	2.96 (1.20)	2.85 (1.28)	3.26 (1.19)	2.43 (1.17)	3.62 (1.15)	55.25^d^	2, 3, 5>4; 3, 5>1, 2
Person capital, mean (SD)								
Self-rated health	3.10 (0.77)	3.12 (0.74)	3.08 (0.77)	3.21 (0.83)	2.88 (0.75)	3.17 (0.94)	33.60^d^	1, 2, 3>4
Long-term health limitation	1.68 (0.47)	1.71 (0.45)	1.67 (0.47)	1.69 (0.46)	1.58 (0.50)	1.61 (0.49)	7.20	—
Depressive symptoms	4.66 (0.88)	4.61 (0.85)	4.77 (0.95)	4.63 (0.81)	4.62 (0.97)	4.80 (0.80)	7.78	—
Self-confidence	6.86 (1.73)	6.74 (1.60)	6.79 (1.98)	7.07 (1.53)	6.85 (1.96)	7.30 (1.91)	151.10^d^	2, 3, 4, 5>1; 3>2, 4; 5>2, 3, 4
Self-image	7.51 (2.21)	7.39 (2.17)	7.59 (2.43)	7.44 (2.01)	7.96 (2.28)	7.63 (2.21)	96.83^d^	2, 3, 4>1; 2, 4, 5>3
Self-rated physical appearance	6.13 (2.10)	6.07 (2.05)	6.15 (2.20)	6.03 (2.01)	6.39 (2.35)	6.66 (1.92)	53.72^d^	4>3
BMI	2.33 (0.72)	2.35 (0.71)	2.30 (0.73)	2.30 (0.74)	2.29 (0.74)	2.44 (0.75)	2.92	—

a Pairwise class differences are based on unadjusted Wald tests; the main patterns remained robust after Benjamini-Hochberg false discovery rate correction.

b Age-stratified sensitivity analyses (<55 vs 55+ years) generally supported the main class characterizations, although some indicator-level differences varied across age groups; detailed results are presented in Table S4 in Multimedia Appendix 1.

c Not available.

d *P*<.05.

#### Class 1: “Neutral Users”

Class 1, the “neutral users,” showed average levels across all 4 forms of capital. In terms of economic capital, they had moderate education levels (significantly higher than class 4, but lower than class 3 and 5) and moderate subjective socioeconomic status (SES; higher than class 2 and 4, but lower than class 3 and 5). Their cultural capital was also moderate, with more engagement in cultural activities and more use of sustainable food than class 4, but less than class 3. They also showed intermediate levels of digital and English language skills (lower than class 3 and 5, but higher than class 4). Regarding social capital, they had a somewhat larger core discussion network than class 4, but smaller than class 3, and received less informal help compared to class 3 and 5. In terms of person capital, this group interestingly showed significantly lower self-image (compared to class 2, 3, and 4) and the lowest self-confidence across all classes.

#### Class 2: “Uninterested Users”

Class 2, the “uninterested users,” were characterized by relatively low economic and cultural capital, although not as low as the “resistant users” in class 4. Their education levels and income were significantly higher than class 4, but lower than class 3 and 5. Their SES was significantly lower than class 1, 3, and 5, but higher than class 4. In terms of cultural capital, they engaged less in holidays abroad, cultural activities, and sustainable food practices compared to class 3. Their digital skills and English language skills were also limited relative to class 3, but still better than those of class 4. For social capital, they reported a smaller core discussion network than class 3 and 5, but larger than class 4. They also received less informal support than class 3 and 5, though more than class 4. Interestingly, their person capital was somewhat mixed. Their self-confidence was lower than in class 3 and 5, but higher than in class 1. In addition, they reported a relatively positive self-image, which was significantly higher than class 1 and 3.

#### Class 3: “Engaged Users”

Class 3, the “engaged users,” showed relatively high levels of capital across all domains. Their economic capital was strong, with significantly higher levels of education (compared to class 1, 2, and 4), SES (compared to class 1, 2, and 4), and income (compared to class 2 and 4). In terms of cultural capital, they reported more frequent participation in cultural activities and holidays abroad, as well as greater sustainable food consumption (compared to class 2 and 4). They also demonstrated better digital skills (compared to class 1, 2, and 4) and English language skills (compared to class 1 and 2). Their social capital was relatively high; they had a larger core discussion network and received more informal support (compared to class 1, 2, and 4). With regard to person capital, they stood out for their high self-confidence (compared to class 1, 2, and 4), although it was still lower than class 5. However, their self-image was significantly lower (compared to class 2, 4, and 5), and they rated their appearance significantly lower than class 4.

#### Class 4: “Resistant Users”

Class 4, the “resistant users,” showed the lowest levels of capital across nearly all indicators. In terms of economic capital, they had significantly lower levels of education (compared to all classes), subjective SES (compared to class 1, 2, and 3), and income (compared to all classes). Their cultural capital was also limited: they participated less in holidays abroad (compared to class 3) and cultural activities (compared to class 1, 3, and 5), reported the least sustainable food consumption (compared to class 1, 3, and 5), and had the weakest digital skills (compared to all classes) and English proficiency (compared to class 1, 2, and 5). Regarding social capital, they had the smallest core discussion network (compared to all classes) and received less informal support than class 2, 3, and 5. Their person capital was also limited, with significantly poorer health (compared to class 1, 2, and 3). However, in contrast, they rated their self-image more positively compared to class 1 and 3. In addition, they rated their appearance more positively compared to class 3.

#### Class 5: “Enthusiastic Users”

The smallest group, class 5, represents the “enthusiastic users.” They showed the highest levels of economic capital, with significantly higher education levels (compared to all other classes), subjective SES (compared to class 1 and 2), and income (compared to class 2 and 4). In terms of cultural capital, they scored higher on digital skills and English proficiency (compared to class 1 and 4) and reported greater sustainable food practices (compared to class 4). Their social capital was also high: they had a larger core discussion network (compared to class 2 and 4) and received more informal support (compared to class 1, 2, and 4). Finally, they stood out in terms of person capital, reporting significantly higher self-confidence (compared to all other classes) and a more positive self-image (compared to class 3).

## Discussion

### Main Findings

This study identified 5 subgroups based on individuals’ acceptance and intended use of digital technologies to support their physical health: neutral users (480/1096, 43.8%), uninterested users (235/1096, 21.4%), engaged users (226/1096, 20.6%), resistant users (102/1096, 9.3%), and enthusiastic users (53/1096, 4.8%). These subgroups differed not only in their level of acceptance and intended use with digital health technologies, but also in their underlying economic, cultural, social, and person capital.

Overall, higher levels of economic, cultural, and social capital were associated with greater acceptance and intended use of digital health technologies. Among all subgroups, enthusiastic users stood out, scoring highest across all forms of capital and showing the greatest openness and enthusiasm toward digital technologies for their physical health. Engaged users also scored high on economic, cultural, and social capital, while resistant and uninterested users scored lower on these capital indicators. This suggests that economic, cultural, and social forms of capital facilitate both acceptance and intended use of digital technologies to support physical health.

A notable finding is the different pattern for person capital. Neutral users, while moderately accepting and using digital health technologies, reported a significantly lower self-image and the lowest self-confidence across all groups. Conversely, uninterested users and resistant users reported a surprisingly positive self-image and self-rated physical appearance, despite facing lower economic, cultural, and social capital.

### Comparison With Prior Work

To explore acceptance and intended use of digital health technologies across the digital divide, many studies have examined the impact of socioeconomic factors. For example, previous latent class analyses have shown that factors such as sex, age, and educational level are relevant in identifying subgroups of digital health technology users [27-29]. Our findings build on these studies by demonstrating that a broader approach, one that incorporates economic, cultural, social, and person capital, can enhance our understanding of who accepts and uses digital technologies to support their physical health.

Our results show that, in general, higher levels of economic, cultural, and social capital were associated with greater acceptance and more frequent tendencies to use digital technologies. This trend was also reflected when looking at individual indicators within each capital form, which aligns with previous studies that focused on some of these specific individual indicators. For example, low digital and language skills (both indicators of cultural capital in this study) have previously been shown to form substantial barriers to digital health engagement [39,58]. Similarly, indicators of social capital, such as the presence of a strong social network, have been previously shown to help individuals overcome barriers to use technologies for their health [24,39]. Our findings support these insights and extend them by integrating these indicators into a broader framework of capital indicators.

Interestingly, person capital emerged as a distinctive and potentially underexplored form of capital in the context of digital health technologies. While neutral users reported moderate levels of economic, cultural, and social capital, they reported the lowest levels of self-confidence and self-image. In contrast, resistant users, despite having limited economic, cultural, and social capital, reported more positive self-perceptions, including higher self-image and more favorable views of their physical appearance. These findings suggest that person capital does not necessarily align with acceptance and intended use of digital health technologies. Although few studies have investigated person capital in relation to digital health technologies, existing research suggests a different dynamic. For example, Mijin et al [59] found that individuals with a more positive self-image may be more strongly influenced by the perceived usefulness of digital technologies in forming favorable attitudes toward adoption. Furthermore, it has been argued that digital technologies are more likely to be adopted when they are congruent with an individual’s self-image [60]. A potential interpretation of our findings is that individuals with higher self-image but low engagement may feel less dependent on digital health technologies or may perceive these technologies as not aligning with how they see themselves. This suggests that strategies aimed at low-adoption groups should not assume low self-image or low perceived capability, but should also consider perceived need and identity congruence. Hence, our findings point to the importance of further exploring how person capital, such as self-image, influences the acceptance and intended use of digital health technologies to assure equitable use across diverse groups of potential users (also see Kadem et al [61] for the importance of personalized and human-centered approaches).

Another finding from our study is that neutral users represented the largest group (480/1096, 43.8% of the sample). These individuals reported average levels of economic, cultural, and social capital, as well as moderate acceptance and intended use of digital health technologies for their physical health. Their neutral attitudes suggest that a substantial portion of the population neither fully adopts nor completely rejects digital health technologies. This aligns with findings, for instance, by Nittas et al [62], who observed that only around 10% of people with multiple sclerosis were active users of health technologies. In our study, a similarly small segment, the enthusiastic users, represented just 4.8% (53/1096) of the sample, followed by the engaged users, who made up 20.6% (226/1096). This group of enthusiastic users, often described in the literature as “early adopters,” consistently reported the highest scores across all forms of capital. Prior research has shown that early adopters tend to be best positioned to benefit from technological innovations, as they often possess greater social resources [63] and higher income [64], aligning with some of our indicators of social and economic capital. Despite their openness and high engagement, the small size of this group highlights a key insight: meaningful use of digital technologies to improve physical health remains the exception rather than the norm. This finding underscores the importance of not only designing for early adopters but also addressing the needs and concerns of the larger, more hesitant middle group.

Similarly, both uninterested and resistant user groups, characterized by low levels of economic, cultural, and social capital, also reported limited acceptance and intended use of digital health technologies. This suggests that digital inequality cannot be fully explained by a lack of access to digital health technologies alone, but instead may also reflect broader economic, cultural, and social influences. Hence, addressing digital health inequalities requires more than just improving acceptance and providing one-time support to use digital health technologies. It calls for an approach that considers all 4 forms of capital for the use of digital health technologies.

In summary, our findings emphasize that the digital divide cannot be fully explained by traditional socioeconomic indicators. Rather, the different forms of capital influence the acceptance and intended use of digital health technologies. In particular, the findings regarding person capital highlight a novel contribution of this study, as person capital did not simply mirror patterns observed for economic, cultural, and social capital. By identifying 5 subgroups, this study provides more insights into who uses digital health technologies for their physical health based on their economic, cultural, social, and person capital. These results can help guide the development of strategies to better support and address the needs of different subgroups. For example, because neutral users represented the largest group, relatively simple forms of support, such as clear onboarding or peer support, may already help this group to become more engaged. For uninterested and resistant users, strategies may need to go beyond improving the technology itself and also address broader barriers, such as limited basic digital skills or limited informal support. More broadly, our findings may also be relevant for the implementation of emerging digital health technologies, including wearable and AI-supported tools, by showing which groups may need additional support to use these technologies and which digital barriers may need to be addressed.

### Limitations and Future Research

This study has several limitations. First, several measurement and modeling choices should be considered when interpreting the findings. The indicators used to measure the 4 types of capital may not fully capture all aspects of each type. For example, we did not include housing wealth and household financial assets in economic capital, nor parental background in cultural capital. In addition, several UTAUT constructs were assessed using single-item measures due to constraints in the available data and to reduce respondent burden. Because single-item measures may be more susceptible to measurement error, observed differences between latent classes on these constructs may underestimate or less precisely capture the true differences between groups. This is specifically relevant for the habit construct among resistant users, where the class-specific mean was at the lowest scale value, suggesting a floor effect and limited variation within this subgroup. The internal consistency of the social influence construct was also slightly below the commonly used threshold of 0.70 (Cronbach α=0.688) [65]. Although this value was close to the threshold and may still be considered reasonable, it may have reduced the measurement precision of this construct. Future research could benefit from using additional indicators and multiple-item scales to improve measurement reliability and to test whether the patterns we identified continue to hold. Finally, the selected 5-class model showed some evidence of local dependence among indicators, as indicated by the remaining maximum BVR. Although the model substantially reduced the maximum BVR compared with the one-class model, some residual associations may not have been fully captured by the latent class model.

Second, although the sample was drawn from a representative Dutch household panel, it was limited to a specific national context. Given the relatively highly developed digital infrastructure and high baseline digital literacy in the Netherlands, the distribution of latent classes may differ in countries where access to and use of digital technologies are more unequally distributed. In addition, although most LISS Core Study data were collected close to the supplementary survey in November 2023, the economic situation data were collected in the June or July 2023 wave. As some capital indicators, such as income, may fluctuate over time, this time lag may have affected how precisely participants’ capital profiles were captured.

Third, our study focused specifically on 4 types of capital and on digital health technologies aimed at supporting physical health. Other potentially relevant factors, such as health literacy and broader digital determinants of health, were not included. Future research could further examine how these capital-based subgroups relate to broader digital determinants of health and how such insights can be translated into implementation strategies for digital health technologies. Future studies could examine whether the identified class differences in technology acceptance and intended use remain robust after accounting for demographic characteristics such as age, sex, and origin, and whether these differences vary across demographic subgroups. Furthermore, because the analyses were cross-sectional, we cannot determine the direction of causality between the capital indicators and acceptance or intended use of digital health technologies. Future longitudinal research is needed to examine whether specific forms of capital shape technology acceptance and intended use, or whether experiences with digital health technologies influence these forms of capital over time.

### Conclusions

This study identified 5 subgroups based on individuals’ acceptance and intended use of digital technologies to support their physical health, ranging from enthusiastic to resistant users. These subgroups differed not only in their digital acceptance and intended use but also in their underlying economic, cultural, social, and person capital. Higher levels of economic, cultural, and social capital were generally associated with greater acceptance and intended use of digital health technologies. However, person capital showed a different pattern, as subgroups with limited resources still reported a positive self-image and self-rated physical appearance. Our findings highlight that users of digital health technologies can be distinguished into subgroups that differ in their levels of economic, cultural, social, and person capital. Addressing digital health inequalities therefore requires strategies that are sensitive to these subgroups and their levels of capital, ensuring that digital health technologies are tailored to the diverse resources and needs across society.

## Supplementary material

10.2196/89446 Multimedia Appendix 1 Supplementary files.

## Abbreviations

AIC: Akaike information criterion
BIC: Bayesian information criterion
BVR: bivariate residual
LISS: Longitudinal Internet Studies for the Social Sciences
LL: log-likelihood
SES: socioeconomic status
UTAUT: unified theory of acceptance and use of technology
