# Supplementary material for: Identifying Subgroups in Acceptance and Intended Use of Digital Technologies and the Role of Economic, Cultural, Social, and Person Capital: Latent Class Analysis
Source: J Med Internet Res. 2026 Sep 24;28:e89446. doi: 10.2196/89446 (PMC13608225; doi:10.2196/89446)
Supplement: Supplementary file 1 [file jmir-v28-e89446-s001.docx]

**Table S1. Items UTAUT constructs**

| **UTAUT construct** | **Items** |
| --- | --- |
| Performance expectancy (PE) | PE1. Using digital technology could support my physical health.  PE2. Using digital technology for my physical health would improve my personal well-being.  PE3. Digital technology can help me improve my physical health. |
| Effort expectancy (EE) | EE1. Digital technology for my physical health would be clear and easy for me to understand.  EE2. Using digital technology for my physical health would be an easy task for me.  EE3. Using digital technology for my physical health would cost me a lot of time and energy. (dropped) |
| Social influence (SI) | SI1. My general practitioner may recommend me to use digital technology for my physical health.  SI2. People around me would recommend me to use digital technology for my physical health.  SI3. People around me regularly use digital technology for their physical health.  SI4. Other people would think badly of me when I use digital technology for my physical health. (dropped) |
| Facilitating conditions (FC) | FC1. I have a smartphone that I can use for digital technology for my health. |
| Hedonic motivation (HM) | HM1. Using digital technology to monitor and improve my physical health is fun. |
| Habit (HT) | HT1. Using digital technology to monitor and improve my physical health is a habit for me. |
| Price value (PV) | PV1. Devices (such as a smart watch) or apps that allow you to examine and improve your health have good value for money. |

**Table S2. Items capital indicators**

| **Capital** | **Resource** | **Items** | **Answer options** |
| --- | --- | --- | --- |
| **Economic capital** |  |  |  |
| Highest educational qualification obtained | LISS Core Study: Background Variables[1] | Highest level of education with diploma | 1 = Primary school, 2 = Vmbo (intermediate secondary education, US: junior high school), 3 = Havo/vwo (higher secondary education / preparatory university education, US: senior high school), 4 = MBO (intermediate vocational education, US: junior college), 5 = HBO (higher vocational education, US: college), 6 = WO (university), 7 = Other |
| Subjective socioeconomic status (SES) | LISS Core Study: Income[2] | If you imagine a 'ladder of life', where the first step represents the worst possible life, and the tenth (top) step the best possible life, on what step would you place yourself? | Scale 1-10 |
| Net household income | LISS Core Study: Background Variables[1] | Net household income in Euros | [Integer] |
| **Cultural capital** |  |  |  |
| International holidays | LISS Core Study: Social Integration and Leisure[3] | How often did you take a holiday abroad over the past 12 months? | 1 = Not once, 2 = One time, 3 = Two times, 4 = Three times, 5 = Four times, 6 = Five times or more |
| Frequency of attending concerts, theatre and museums | LISS Core Study: Social Integration and Leisure[3] | How often did you visit these performances or concerts over the past 12 months?   - A theatre performance, number of visits over the past 12 months - A museum, number of visits over the past 12 months - A concert/festival of pop, rock, jazz, rap, hiphop | 0 = 0 times, 1 = 1 time, 2 = 2 to 3 times, 3 = 4 to 11 times, 4 = 12 times or more |
| Use of sustainable food | Additional survey[4] | In the past 12 months, have you purchased the following products?   - Fairtrade coffee or tea - Organic vegetables - Free-range meat - Free-range or organic eggs | 0 = No, 1 = Yes |
| Basic digital skills | Additional survey[4] | - The first search result is always the best source of information. - Everyone receives the same information when searching online for the same things. - Some people make money by spreading fake news on the internet. - Before digitally sharing a photo clearly showing a friend, you must always ask for permission first. - Hurtful comments online hurt less than saying them to someone's face. | 1 = Definitely true, 2 = Definitely false, 3 = I don't know, 4 = I don't understand the question |
| English language proficiency | Additional survey[4] | Do you speak English? | 1 = No, 2 = A little, 3 = Fairly, 4 = Well, 5 = Native speaker |
| **Social capital** |  |  |  |
| Contact with family members (not living in the same household) | Additional survey[4] | How often do you have contact with the following groups? Family members (not living with you) | 1 = At least once a week, 2 = Once every 2 weeks, 3 = Once a month, 4 = Less than once a month, 5 = (almost) never, 6 = Not applicable, 7 = Don't know |
| Contact with close friends | Additional survey[4] | How often do you have contact with the following groups? Friends or very close acquaintances | 1 = At least once a week, 2 = Once every 2 weeks, 3 = Once a month, 4 = Less than once a month, 5 = (almost) never, 6 = Not applicable, 7 = Don't know |
| Contact with neighbors and community members | Additional survey[4] | How often do you have contact with the following groups? Neighbors and community members | 1 = At least once a week, 2 = Once every 2 weeks, 3 = Once a month, 4 = Less than once a month, 5 = (almost) never, 6 = Not applicable, 7 = Don't know |
| Contact with colleagues outside working hours | Additional survey[4] | How often do you have contact with the following groups? Colleagues outside working hours | 1 = At least once a week, 2 = Once every 2 weeks, 3 = Once a month, 4 = Less than once a month, 5 = (almost) never, 6 = Not applicable, 7 = Don't know |
| Size of core discussion network | Additional survey[4] | Do you know someone to whom you can ask for help who...  - can help with household tasks (carpentry, painting) or repairs  - can do groceries if you are ill  - can offer a place to stay if you need to leave your home temporarily  - can give advice about a conflict at work  - can give advice about a family conflict | 0 = No, 1 = Yes |
| Informal support | Additional survey[4] | How many people (not living with you) did you discuss important personal matters with offline or online in the past six months? | 1 = None, 2 = 1 person, 3 = 2 or 3 persons, 4 = 4 or 5 persons, 5 = More than 5 persons |
| **Person capital** |  |  |  |
| Self-rated health | LISS Core Study: Health[5] | How would you describe your health, generally speaking? | 1 = Poor, 2 = Moderate, 3 = Good, 4 = Very good, 5 = Excellent |
| Long-term health limitation | LISS Core Study: Health[5] | Do you suffer from a long-standing disease, affliction or handicap, or do you suffer from the consequences of an accident? | 1 = Yes, 2 = No |
| Depressive symptoms (MH) | LISS Core Study: Health[5] | - I felt very anxious. - I felt so down that nothing could cheer me up. - I felt calm and peaceful. - I felt depressed and gloomy. - I felt happy. | 1 = Never, 2 = Seldom, 3 = Sometimes, 4 = Often, 5 = Mostly, 6 = Continuously |
| Self-confidence | Additional survey[4] | I have a lot of self-confidence. | Scale 1-10 (1=Strongly agree, 10=Strongly disagree) |
| Self-image | Additional survey[4] | I have a negative image of myself. | Scale 1-10 (1=Strongly agree, 10=Strongly disagree) |
| Self-rated physical appearance | Additional survey[4] | I find my appearance just right as it is. | Scale 1-10 (1=Strongly agree, 10=Strongly disagree) |
| Body Mass Index (BMI) | LISS Core Study: Health[5, 6] | Height, weight | [Integer], BMI = weight / (height * height in meters), score: < 18.5 = 2, 18.5–25 = 3, 25–30 = 2, ≥ 30 = 1 |

1.	LISS Data Archive / Background Variables / English 2023 November [database on the Internet]. 2022. Available from: <https://doi.org/10.57990/qn3k-as78>.

2.	LISS Data Archive / Economic Situation: Income: Wave 16 [database on the Internet]. 2023. Available from: <https://doi.org/10.57990/1gr4-bf42>.

3.	LISS Data Archive / Background Variables / Social Integration and Leisure: Wave 16 [database on the Internet]. 2024. Available from: <https://doi.org/10.57990/2gze-ha88>.

4.	Vrooman C, Boelhouwer J, Iedema J, Van der Torre A. Eigentijdse ongelijkheid. De postindustriële klassenstructuur op basis van vier typen kapitaal. Sociaal en Cultureel Planbureau. 2023.

5.	LISS Data Archive / Health: Wave 16 [database on the Internet]. 2024. Available from: <https://doi.org/10.57990/evk0-mk06>.

6.	Voedingscentrum. BMI berekenen. 2026; Available from: <https://www.voedingscentrum.nl/bmi>.

**Table S3. Items sociodemographic characteristics**

| **Characteristics** | **Items** | **Answer options** |
| --- | --- | --- |
| **Gender** | Gender | 1 = Male, 2 = Female, 3 = Other |
| **Age** | Age | 1 = 14 years and younger, 2 = 15 - 24 years, 3 = 25 - 34 years, 4 = 35 - 44 years, 5 = 45 - 54 years, 6 = 55 - 64 years, 7 = 65 years and older |
| **Origin** | Origin | 0 = Dutch background, 101 = First generation foreign, Western background, 102 = First generation, non-western background, 201 = Second generation, Western background, 202 = Second generation, non-western background, 999 = Origin unknown or part of the information unknown (missing values) |

**Table S4. Age-stratified sensitivity analysis of capital indicators across the five latent classes**

| **Capital indicator** | **Age group** | **Overall within age group** | **Class 1 ‘Neutral users’** | **Class 2 ‘Uninterested users’** | **Class 3 ‘Engaged users’** | **Class 4 ‘Resistant users’** | **Class 5 ‘Enthusiastic’ users** | **Wald test** |
| --- | --- | --- | --- | --- | --- | --- | --- | --- |
| **Class size, n (%)** |  |  |  |  |  |  |  |  |
|  | Full sample | 1096 (100.0%) | 480 (43.8%) | 235 (21.4%) | 226 (20.6%) | 102 (9.3%) | 53 (4.8%) |  |
|  | <55 years | 617 (56.3%) | 288 (46.7%) | 105 (17.0%) | 155 (25.1%) | 34 (5.5%) | 35 (5.7%) |  |
|  | 55+ years | 479 (43.7%) | 192 (40.1%) | 130 (27.1%) | 71 (14.8%) | 68 (14.2%) | 18 (3.8%) |  |
| **Economic capital**, *M (SD)* |  |  |  |  |  |  |  |  |
| Highest educational qualification obtained | <55 years | 3.35 (0.76) | 3.27 (0.76) | 3.29 (0.80) | 3.60 (0.63) | 2.76 (0.90) | 3.68 (0.53) | 72.30* |
|  | 55+ years | 3.04 (0.91) | 3.09 (0.87) | 3.04 (0.91) | 3.25 (0.82) | 2.62 (1.03) | 3.22 (0.73) | 42.90* |
| Subjective socioeconomic status (SES) | <55 years | 7.02 (1.39) | 7.02 (1.33) | 6.94 (1.34) | 7.27 (1.26) | 6.31 (2.04) | 7.15 (1.53) | 250.49* |
|  | 55+ years | 7.23 (1.28) | 7.30 (1.19) | 7.19 (1.30) | 7.47 (1.02) | 6.81 (1.57) | 7.33 (1.63) | 160.52* |
| Net household income | <55 years | 4392.84 (2423.30) | 4488.15 (2443.93) | 4247.07 (2231.86) | 4406.83 (2532.32) | 3180.0 (1384.13) | 5045.06 (2723.13) | 7.71 |
|  | 55+ years | 3581.28 (1923.88) | 3614.82 (1938.62) | 3474.73 (1928.05) | 4293.05 (1880.07) | 2762.87 (1460.77) | 4281.12 (2303.77) | 12.58* |
| **Cultural capital**, *M (SD)* |  |  |  |  |  |  |  |  |
| International holidays | <55 years | 2.36 (1.26) | 2.29 (1.25) | 2.38 (1.29) | 2.63 (1.34) | 2.04 (1.15) | 2.29 (1.08) | 130.53* |
|  | 55+ years | 2.06 (1.26) | 2.21 (1.35) | 1.81 (1.13) | 2.27 (1.23) | 1.74 (0.97) | 2.67 (1.72) | 123.65* |
| Frequency of attending concerts, theatre and museums | <55 years | 0.47 (0.45) | 0.47 (0.44) | 0.42 (0.46) | 0.55 (0.45) | 0.26 (0.35) | 0.50 (0.50) | 13.04* |
|  | 55+ years | 0.42 (0.48) | 0.42 (0.49) | 0.39 (0.45) | 0.58 (0.57) | 0.30 (0.45) | 0.47 (0.33) | 5.92 |
| Use of sustainable food | <55 years | 1.89 (1.43) | 1.81 (1.40) | 1.70 (1.47) | 2.23 (1.41) | 1.14 (1.31) | 2.43 (1.36) | 28.94* |
|  | 55+ years | 2.01 (1.36) | 2.11 (1.34) | 1.94 (1.31) | 2.31 (1.38) | 1.43 (1.33) | 2.33 (1.37) | 28.53* |
| Basic digital skills | <55 years | 0.81 (0.28) | 0.77 (0.31) | 0.81 (0.26) | 0.89 (0.20) | 0.66 (0.34) | 0.87 (0.19) | 283.62* |
|  | 55+ years | 0.71 (0.30) | 0.72 (0.29) | 0.72 (0.30) | 0.83 (0.23) | 0.50 (0.34) | 0.81 (0.18) | 91.00* |
| English language proficiency | <55 years | 3.31 (0.77) | 3.19 (0.78) | 3.41 (0.77) | 3.51 (0.63) | 2.85 (1.08) | 3.49 (0.51) | 278.06* |
|  | 55+ years | 2.73 (0.87) | 2.80 (0.86) | 2.65 (0.83) | 2.97 (0.81) | 2.31 (0.91) | 3.17 (0.92) | 99.56* |
| **Social capital,** *M (SD)* |  |  |  |  |  |  |  |  |
| Contact with family members (not living in the same household) | <55 years | 1.63 (1.02) | 1.62 (1.03) | 1.70 (1.07) | 1.53 (0.94) | 2.06 (1.22) | 1.60 (0.98) | 36.63* |
|  | 55+ years | 1.58 (1.08) | 1.60 (1.12) | 1.56 (1.09) | 1.52 (0.99) | 1.73 (1.11) | 1.17 (0.71) | 87.50* |
| Contact with close friends | <55 years | 1.68 (1.03) | 1.72 (1.03) | 1.75 (1.14) | 1.56 (0.97) | 1.69 (0.90) | 1.69 (1.05) | 71.16* |
|  | 55+ years | 1.84 (1.08) | 1.82 (1.09) | 1.85 (1.08) | 1.77 (1.02) | 2.11 (1.18) | 1.22 (0.43) | 83.80* |
| Contact with neighbors and community members | <55 years | 2.85 (1.42) | 2.78 (1.41) | 3.04 (1.32) | 2.91 (1.47) | 2.73 (1.60) | 2.65 (1.45) | 13.74 |
|  | 55+ years | 2.38 (1.43) | 2.34 (1.43) | 2.26 (1.35) | 2.56 (1.43) | 2.61 (1.59) | 2.06 (1.25) | 11.10 |
| Contact with colleagues outside working hours | <55 years | 3.37 (1.41) | 3.38 (1.36) | 3.33 (1.35) | 3.50 (1.49) | 3.18 (1.59) | 3.03 (1.38) | 19.98 |
|  | 55+ years | 3.66 (1.34) | 3.72 (1.41) | 3.80 (1.28) | 3.23 (1.22) | 3.91 (1.11) | 3.18 (1.54) | 55.13* |
| Size of core discussion network | <55 years | 3.94 (1.54) | 3.81 (1.60) | 3.83 (1.63) | 4.33 (1.24) | 3.09 (1.80) | 4.49 (1.07) | 200.47* |
|  | 55+ years | 2.85 (1.50) | 2.83 (1.45) | 2.91 (1.53) | 3.20 (1.51) | 2.31 (1.45) | 3.44 (1.50) | 37.00* |
| Informal support | <55 years | 3.13 (1.21) | 3.05 (1.17) | 3.08 (1.19) | 3.38 (1.21) | 2.32 (1.15) | 3.71 (1.23) | 31.09* |
|  | 55+ years | 2.78 (1.24) | 2.82 (1.22) | 2.67 (1.33) | 3.00 (1.12) | 2.48 (1.19) | 3.44 (0.98) | 91.74* |
| **Person capital**, *M (SD)* |  |  |  |  |  |  |  |  |
| Self-rated health | <55 years | 3.26 (0.81) | 3.22 (0.76) | 3.34 (0.88) | 3.34 (0.87) | 3.08 (0.80) | 3.38 (0.87) | 27.49* |
|  | 55+ years | 2.95 (0.71) | 3.00 (0.69) | 2.94 (0.67) | 3.02 (0.74) | 2.80 (0.72) | 2.87 (0.99) | 115.29* |
| Long-term health limitation | <55 years | 1.82 (0.39) | 1.82 (0.38) | 1.81 (0.39) | 1.81 (0.39) | 1.85 (0.37) | 1.71 (0.46) | 1.65 |
|  | 55+ years | 1.55 (0.50) | 1.57 (0.50) | 1.60 (0.49) | 1.52 (0.50) | 1.47 (0.50) | 1.47 (0.52) | 3.58 |
| Depressive symptoms | <55 years | 4.50 (0.87) | 4.46 (0.85) | 4.57 (1.03) | 4.53 (0.81) | 4.45 (0.98) | 4.50 (0.74) | 6.40 |
|  | 55+ years | 4.87 (0.84) | 4.84 (0.82) | 4.93 (0.85) | 4.84 (0.79) | 4.71 (0.96) | 5.39 (0.54) | 8.16 |
| Self-confidence | <55 years | 6.71 (1.75) | 6.64 (1.60) | 6.51 (2.11) | 6.90 (1.61) | 6.79 (2.35) | 6.94 (1.63) | 197.98* |
|  | 55+ years | 7.04 (1.69) | 6.88 (1.60) | 7.02 (1.84) | 7.42 (1.28) | 6.88 (1.75) | 8.00 (2.25) | 145.19* |
| Self-image | <55 years | 7.09 (2.20) | 7.05 (2.15) | 7.03 (2.61) | 7.09 (1.99) | 7.50 (2.53) | 7.20 (1.83) | 195.61* |
|  | 55+ years | 8.04 (2.11) | 7.89 (2.10) | 8.05 (2.18) | 8.20 (2.13) | 8.19 (2.13) | 8.44 (2.68) | 201.08* |
| Self-rated physical appearance | <55 years | 5.84 (2.07) | 5.86 (2.04) | 5.74 (2.28) | 5.81 (1.96) | 5.85 (2.49) | 6.03 (1.71) | 83.22* |
|  | 55+ years | 6.51 (2.08) | 6.36 (2.03) | 6.47 (2.08) | 6.49 (2.05) | 6.66 (2.25) | 7.89 (1.75) | 75.86* |
| Body Mass Index (BMI) | <55 years | 2.40 (0.75) | 2.38 (0.75) | 2.42 (0.77) | 2.33 (0.78) | 2.56 (0.58) | 2.60 (0.68) | 5.42 |
|  | 55+ years | 2.26 (0.70) | 2.32 (0.67) | 2.23 (0.69) | 2.25 (0.68) | 2.19 (0.77) | 2.21 (0.80) | 6.05 |

^*^*p* < 0.05.
